# Hereditary angioedema: on-demand treatment and long-term prophylaxis—a global reality

**DOI:** 10.1093/cei/uxag043

**Published:** 2026-07-25

**Authors:** Cassim Akhoon, Sorena Kiani-Alikhan

**Affiliations:** Royal Free London NHS Foundation Trust, Department of Immunology, London, UK; Royal Free London NHS Foundation Trust, Department of Immunology, London, UK

**Keywords:** complement, skin, human

## Abstract

Hereditary angioedema (HAE) is a rare disorder of recurrent swellings of the subcutaneous and/or mucosal tissues due to defects in the contact system regulation of bradykinin production. The swellings can be disfiguring, cause debilitating abdominal pain, or be fatal with laryngeal obstruction. Over the last decade, there have been immense successes in management of HAE owing to the development of drugs targeting the contact system. This review focuses on management of HAE due to C1-esterase inhibitor (C1-INH) deficiency/dysfunction, including a global perspective which is under-represented in the literature. HAE with normal C1-INH is outside the scope of this article. The review provides summaries of clinical trials that have led to licensing of new HAE medications and discusses access to these modern drugs from a global perspective.

## Teaser

Hereditary angioedema (HAE) is a rare disorder of recurrent swellings of the subcutaneous and/or mucosal tissues due to defects in the contact system regulation of bradykinin production. This review focuses on management of HAE due to C1-esterase inhibitor deficiency/dysfunction including a global perspective which is under-represented in the literature.

## Introduction

**Figure 1:**
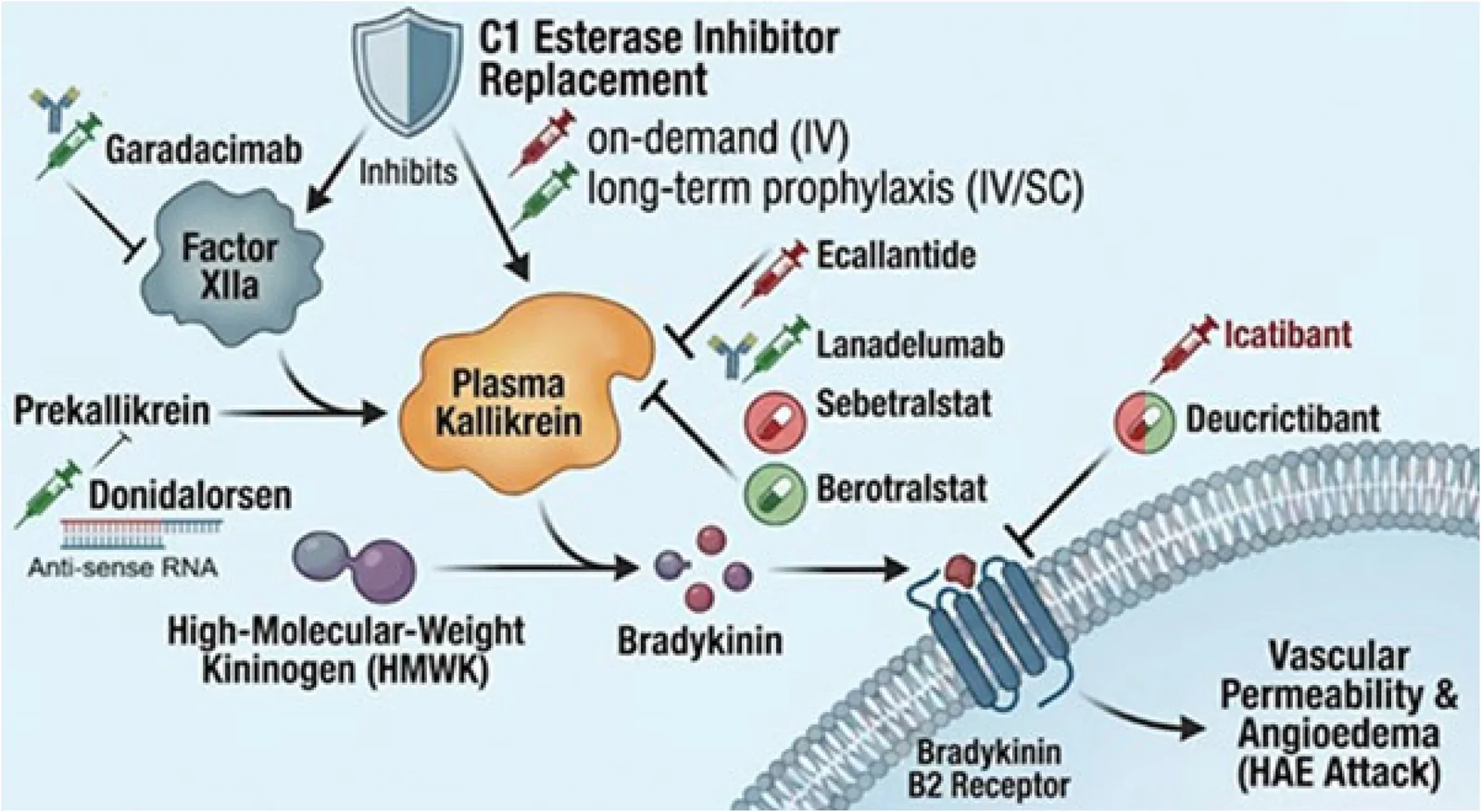
Bradykinin pathway in hereditary angioedema (HAE) and therapeutic targets. Schematic of the kallikrein–kinin (contact) system cascade leading to angioedema and the sites of action of available and emerging therapies. Contact system activation generates factor XIIa (FXIIa), which converts prekallikrein to plasma kallikrein. Plasma kallikrein cleaves high-molecular-weight kininogen (HMWK) to produce bradykinin. Bradykinin binding to endothelial bradykinin B2 receptors increases vascular permeability, resulting in angioedema (HAE attack). Therapeutic agents interrupt this pathway at defined targets: C1-esterase inhibitor (C1-INH) replacement inhibits FXIIa and plasma kallikrein; FXIIa inhibition (e.g. garadacimab) prevents kallikrein activation; plasma kallikrein inhibition (e.g. ecallantide; oral kallikrein inhibitors such as sebetralstat and berotralstat) reduces bradykinin generation; B2 receptor antagonism (e.g. icatibant; oral B2 antagonists such as deucrictibant) blocks bradykinin signalling; and prekallikrein suppression (e.g. donidalorsen) reduces kallikrein generation upstream. Therapies are colour-coded by clinical role (red, on-demand; green, long-term prophylaxis). Deucrictibant is shown with dual colour-coding to reflect development of immediate-release formulations for on-demand treatment and extended-release formulations for long-term prophylaxis. Route of administration is indicated by icon type (syringe, injectable; pill, oral). An immunoglobulin is displayed beside the two therapies which are monoclonal antibodies, and RNA beside that which is anti-sense.

Hereditary angioedema (HAE) is a rare, predominantly autosomal dominant disorder characterized by recurrent, unpredictable non-pitting oedema of the subcutaneous and/or mucosal tissues. Attacks can be debilitating, causing disfiguring swelling or severe abdominal pain, and may be fatal with laryngeal obstruction, necessitating rapid and effective treatment. Most cases arise from C1-esterase inhibitor (C1-INH) deficiency (type 1) or dysfunction (type 2), leading to excess bradykinin generation (Fig. 1) [1]. Consequently, attacks do not respond to antihistamines, corticosteroids, or adrenaline [2].

Long-term prophylaxis (LTP) is important for overall disease control, while on-demand therapy remains central for managing breakthrough attacks [1]. Early on-demand treatment shortens attack duration, may reduce complications, and supports home management through self-administration [3].

Over the past two decades, acute treatment has evolved from reliance on supportive care and fresh-frozen plasma (FFP) [4] to targeted agents that replace C1-INH or inhibit bradykinin generation/signalling; LTP has moved beyond attenuated androgens (AAs) to disease-specific agents [5]. However, timely diagnosis and access to these advances remain unevenly distributed, with substantial global variation, particularly in low- and middle-income countries (LMICs) [6–8].

This is a narrative review focused on HAE due to C1-INH deficiency or dysfunction, considered with a global perspective. HAE with normal C1-INH is outside the scope of this article. The review is intended as a clinically orientated synthesis of contemporary therapeutic evidence and considerations on global access. Evidence was prioritized from pivotal and extension trial data, major international guidelines, real-world registry/audit data, and studies evaluating geographic disparities in access to therapies and participation in clinical trials. Where efficacy outcomes are presented, they are reported using the endpoint definitions used by each study programme and should be interpreted descriptively rather than as indirect comparisons between therapies.

## Currently available on-demand treatment

A range of on-demand therapies are available for the treatment of acute HAE attacks, although availability varies substantially by region. Direct head-to-head comparisons are limited because pivotal studies used different endpoints, instruments and analysis approaches (e.g. time to onset/beginning of symptom relief, time to ≥50% symptom reduction, composite symptom scores at fixed time points, or treatment outcome scores), and attack-site mix and rescue rules also varied between studies. In addition, FFP remains an off-label fall-back option where targeted therapies are unavailable. The section below reports each programme’s primary (or most comparable) efficacy endpoint and, where relevant, fixed-time-point symptom score outcomes. These figures are descriptive and should not be interpreted as head-to-head comparative efficacy. A summary is provided in Table 1.

**Table 1:** On-demand therapies for hereditary angioedema due to C1-esterase inhibitor deficiency (HAE-C1-INH): route of administration, potential for self-administration, dosing (including repeat dosing), and pivotal trial efficacy endpoints.

Therapy	Mechanism of action	Route	Potential for self-administration	Dose	Repeat dosing if inadequate response	Efficacy (pivotal endpoint + time/score)
pdC1-INH	C1-INH replacement	IV	Possible with training	20 IU/kg	Max 2 doses/24 h	Median time to onset of symptom relief 0.5 h (placebo 1.5 h)
rhC1-INH	C1-INH replacement	IV	Possible with training	50 IU/kg Max 4200 IU	Max 2 doses/24 h	Median time to beginning of symptom relief 90 min (placebo 152 min)
Icatibant	B2 receptor antagonism	SC	Commonly feasible	30 mg	Max 3 doses/24 h; minimum 6-hourly interval	Median time to ≥50% reduction in symptom severity 2.0 h (placebo 19.8 h)
Ecallantide	Kallikrein inhibition	SC	No	30 mg	Max 2 doses/24 hours	Median treatment outcome score (TOS) at 4 h 50.0 (placebo 0.0); median change in Mean Symptom Complex Severity (MSCS) at 4 h −1.00 (placebo −0.50).
Sebetralstat	Kallikrein inhibition	Oral	Yes	300 mg	Repeat after 3 h. Max 2 doses/24 h	Median time to onset of symptom relief 1.61 h (IQR 0.78–7.04) for 300 mg; placebo 6.72 h
**Fresh Frozen Plasma (FFP) (off-label)**	Non-specific replacement incl. C1-INH	IV	No	Variable (∼10–20 ml/kg)	Variable	N/A

Doses and repeat dosing reflect typical adult/adolescent regimens from product labelling and/or commonly used institutional protocols; clinical judgement is required, particularly for airway attacks, delayed presentation, or inadequate response. Availability varies by region; fresh frozen plasma (FFP) is included as an off-label fallback option where approved targeted therapies are unavailable.

(i) Efficacy figures are reported using the primary (or most commonly cited) endpoint from each pivotal programme and are presented for orientation only; endpoints, instruments, assessment schedules, attack-site mix, rescue rules, and statistical handling differ between trials, so cross-trial comparisons should be interpreted cautiously and do not constitute head-to-head evidence. (ii) Time-to-event values are shown as medians unless otherwise stated; for Ecallantide, validated fixed-time-point symptom outcomes (TOS/MSCS) at 4 hours are shown rather than a single time-to-relief measure. (iii) For Sebetralstat, the table reports KONFIDENT trial values for 300 and 600 mg (IQR shown where reported); dosing in clinical practice follows approved local labelling. (iv) FFP lacks landmark randomized placebo-controlled trial data in HAE-C1-INH; evidence is observational/real-world and practice is centre-specific.

SC, subcutaneous; TOS, Treatment Outcome Score.

### C1-INH—human plasma-derived

Plasma-derived C1-INH (pdC1-INH) products provide functional C1-INH to down-regulate FXIIa and plasma kallikrein activity, reducing bradykinin generation. These agents support early treatment and, in some health systems, home administration, but require intravenous (IV) access and aseptic reconstitution. Product indications and availability vary by jurisdiction.

The efficacy of acute treatment has been demonstrated for more than one pdC1-INH product. In a randomized, double-blind, placebo-controlled crossover trial, nanofiltered C1-INH subsequently marketed as Cinryze, shortened the median time to onset of unequivocal relief compared with placebo (2 vs >4 hours) of acute HAE attacks [9]. Berinert was evaluated in the International Multicentre Prospective Angioedema C1-INH Trial-1 (IMPACT-1), which was a randomized, double-blind, placebo-controlled trial demonstrating that pdC1-INH (20 IU/kg) significantly shortened the median time to onset of symptom relief for acute abdominal/facial attacks (0.5 hours) compared with placebo (1.5 hours) [10]. The IMPACT-2 extension study confirmed the safety and efficacy of treating multiple successive attacks [11, 12].

### C1-INH—recombinant (Conestat alfa; Ruconest)

Recombinant human C1-INH (rhC1-INH) is administered intravenously and has the same downstream mechanism as pdC1-INH. As a recombinant product produced in transgenic rabbits, rhC1-INH avoids reliance on human plasma and eliminates the theoretical risk of human blood-borne pathogen transmission. It has the added advantage of a long shelf-life (up to 4 years, whilst pdC1-INH products typically are 2–3 years depending on product and jurisdiction). However, known or suspected rabbit allergy is a labelled contraindication, not merely a theoretical risk. During clinical development, there was a single case of anaphylaxis in the Phase 1 study in a participant with previously undisclosed rabbit allergy and substantially elevated anti-rabbit epithelium immunoglobulin E (IgE) [13]. In a retrospective immunosafety analysis, four additional subjects with lower-level pre-existing anti-rabbit epithelium IgE tolerated rhC1-INH, and repeated administration did not appear to induce or boost clinically relevant IgE responses. Subsequent clinical programme analyses and post-marketing surveillance have not identified a broader signal of severe or serious anaphylaxis in rabbit-allergic patients, but this should be interpreted in the context of labelled avoidance and likely limited intentional exposure of such patients [14].

Two randomized, double-blind, placebo-controlled trials supported efficacy in acute attacks [15], and a subsequent Phase 3 trial (C1 1310) reported a significantly shorter median time to beginning of symptom relief with rhC1-INH 50 IU/kg (maximum of 4200 IU) compared with placebo (90 vs 152 minutes) [16]. Furthermore, rhC1-INH has a shorter elimination half-life (∼2 hours) [17] than plasma-derived C1-INH (e.g. Berinert median ∼36.1 hours) [18], which has been cited as a potential contributor to symptom recurrence and re-dosing, although recurrence appears uncommon and most attacks are controlled with a single 50 U/kg dose [19].

### Icatibant (Firazyr)

Icatibant is a synthetic decapeptide and selective bradykinin B2 receptor antagonist administered subcutaneously. By competitively blocking B2 receptors, it prevents bradykinin signalling that drives increased vascular permeability and vasodilation during attacks.

In the FAST-3 randomized, double-blind, placebo-controlled Phase 3 trial, Icatibant (30 mg; with up to three injections per attack in extension phase) significantly reduced the median time to 50% symptom reduction compared with placebo (2.0 vs 19.8 hours) [20]. Earlier trials also supported benefit; FAST-2 demonstrated superiority of Icatibant compared with oral tranexamic acid (2.0 vs 12.0 hours) [21].

### Ecallantide (Kalbitor)

Ecallantide is a recombinant protein that functions as a potent, selective, reversible inhibitor of plasma kallikrein. It reduces cleavage of high-molecular-weight kininogen (HMWK) and prevents bradykinin generation during an attack.

Phase 3 randomized, double-blind, placebo-controlled trials (EDEMA3 and EDEMA4) [22, 23] demonstrated significantly greater improvement in validated, HAE-specific outcomes (Treatment Outcome Score and Mean Symptom Complex Severity) at 4-hour post-dosing (30 mg) versus placebo. The time to significant improvement with Ecallantide was 165  versus 240 minutes with placebo. However, hypersensitivity reactions, including anaphylaxis, were observed in a small proportion of patients; therefore, its administration is restricted to only be used by healthcare professionals with appropriate emergency capability [24].

### Sebetralstat (Ekterly)

Sebetralstat is a small-molecule plasma kallikrein inhibitor and is the first approved oral on-demand therapy for acute HAE attacks. By removing the need for injections, oral therapy may reduce treatment delays driven by administration hesitance and could improve feasibility of early treatment.

In the KONFIDENT phase 3 randomized, placebo-controlled crossover trial, sebetralstat (300 or 600 mg) achieved significantly faster time to beginning of symptom relief than placebo across attack locations (1.61 hours with 300 mg, 1.79 hours with 600 mg, and 6.72 hours with placebo), with a safety profile comparable to placebo and no serious treatment-related adverse events [25]. The extent of global impact will ultimately depend on regulatory approvals, pricing, procurement pathways, and supply stability.

### Fresh frozen plasma

FFP is an IV infusion of donor plasma containing endogenous C1-INH and has historically been used as a pragmatic on-demand option where specific HAE therapies are unavailable, with variable dosing practices. As FFP also contains contact system substrates (including prekallikrein and HMWK) there is a theoretical, and occasionally reported, risk of transient symptom worsening if substrate provision precedes net inhibitory effect [4]. Unlike targeted agents, FFP lacks landmark randomized, placebo-controlled evidence in HAE. Its use is supported by observational data and is typically positioned as a fall-back when approved on-demand therapies are inaccessible [1, 26]. Practical limitations include blood product handling requirements, slower time-to-administration, and standard transfusion risks (e.g. reactions, TRALI/TACO, infection, and potential thromboembolic risks) [27].

### Investigational: Deucrictibant immediate-release (IR; PHVS416)

Deucrictibant is an investigational, small-molecule bradykinin B2 receptor antagonist with a mechanism analogous to Icatibant but delivered orally. The phase 2 RAPIDe-1 study supported proof-of-concept for rapid symptom improvement and attack resolution versus placebo for acute HAE attacks [28, 29]. Longer-term on-demand use is being evaluated in an open-label extension (RAPIDe-2) [30], and a pivotal phase 3 study (RAPIDe-3; NCT06343779) is evaluating an oral soft-capsule formulation for on-demand treatment to define efficacy across attack sites and broader patient populations [31].

### Synthesis of evidence for on-demand treatment

Taken together, the evidence for on-demand treatment supports early treatment of all HAE attacks with targeted therapy, but interpretation is limited by the absence of direct head-to-head comparisons and by heterogeneity in endpoints, attack-site mix, rescue medication rules set by individual trials, and assessment instruments across trial programmes. Most pivotal studies enrolled predominantly adult and adolescent populations, with more limited paediatric evidence, particularly in younger children. In well-resourced settings, treatment choice is therefore usually driven less by proven comparative efficacy and more by practical considerations, including speed of access, route of administration, patient confidence with self-treatment, previous response, safety profile, co-morbidities, local licensing, and reimbursement. IV C1-INH products remain particularly relevant and are still considered gold standard for pregnancy, breastfeeding, paediatric use, or if there are specific contraindications. Subcutaneous Icatibant offers practical self-administration but is associated with injection-site reactions and still requires willingness to inject. Ecallantide is less compatible with self-treatment because of hypersensitivity risk and healthcare-professional administration requirements. Oral on-demand agents may reduce delays caused by injection hesitancy, venous access, reconstitution, cold-chain requirements, or lack of trained personnel; however, their global impact will depend on affordability, registration, procurement and reliable supply. Key research gaps include active-comparator trials, harmonized outcome definitions, more robust paediatric data, evidence in pregnancy and breastfeeding, real-world effectiveness studies across diverse health systems and cost-effectiveness analyses that include settings where FFP remains the only realistic fallback.

## Currently available long-term prophylactic agents

LTP aims to reduce HAE attack frequency and severity, improve health-related quality of life, and reduce reliance on emergency care. Contemporary guidance increasingly supports individualized LTP selection based on disease burden (attack frequency and severity), patient preference, co-morbidities, and feasibility of administration (self-injection vs oral therapy), while emphasizing that all patients should still maintain reliable access to effective on-demand therapy for breakthrough attacks. Mechanistically, modern LTP mirrors on-demand therapy by targeting the kallikrein–kinin pathway, but with sustained suppression rather than episodic interruption [1, 2]. Attack-free proportions and number of attacks requiring on-demand therapy are reported as defined in the original trials (including their specified analysis windows); these outcomes are not fully harmonized across programmes and may be influenced by local practice and treatment-seeking behaviour [5]. This is summarized in Table 2.

**Table 2:** Long-term prophylactic therapies for HAE-C1-INH: route of administration, potential for self-administration, typical dosing regimens, and pivotal trial efficacy outcomes.

Therapy	Mechanism/target	Route	Potential for self-administration	Typical regimen	Efficacy in pivotal trial (primary endpoint + key secondaries)
pdC1-INH (Cinryze)	C1-INH replacement	IV	Possible with training	1000 U twice weekly (commonly)	Attack frequency 6.26/12 weeks vs placebo 12.72/12 weeks; attacks requiring on-demand therapy 4.7 vs 15.4 (12-week periods). Attack-free rates not reported.
pdC1-INH (HAEGARDA)	C1-INH replacement	SC	Yes (after training)	60 IU/kg twice weekly	Mean attacks/month 0.52 vs 4.03 (SC C1-INH vs placebo); attacks requiring on-demand therapy 35 vs 358 (SC C1-INH vs placebo); attack-free ∼40%;
Lanadelumab	Plasma kallikrein inhibition	SC	Yes (after training)	300 mg every 2–4 weeks	Model-based attack rate 0.26–0.53/month vs placebo 1.97/month; attacks requiring on-demand therapy 0.26–0.38 month vs 1.58/month placebo; attack-free over 26 weeks 31–44% vs 2% placebo
Berotralstat	Plasma kallikrein inhibition	Oral	Yes	150 mg once daily	Attack rate 1.31–1.65/month vs placebo 2.35/month; attacks requiring on-demand therapy 1.04/month vs 2.05/month (150 mg vs placebo). Attack-free 7.5% vs 5.0% (150 mg vs placebo; 24 weeks) inferred from data.
Garadacimab	FXIIa inhibition	SC	Yes (after training)	400 mg loading dose, 200 mg monthly maintenance	Time-normalised attack rate 0.27/month vs placebo 2.01/month; attacks requiring on-demand therapy 0.23/month vs 1.86/month; attack-free 62% vs 0% (6 months)
Donidalorsen	Prekallikrein mRNA degradation	SC	Yes (after training)	80 mg every 4–8 weeks	Mean attack rate per 4 weeks 0.44 vs placebo 2.26 (q4w); 1.02 with q8w; attacks requiring on-demand therapy 0.15 vs 1.8 (Weeks 4–24); attack-free 53% vs 9% (Weeks 4–24)
Attenuated androgens	↑ hepatic C1-INH synthesis (non-targeted)	Oral	Yes	Variable; ‘lowest effective dose’	Historic controlled data (small, heterogeneous endpoints): attacks in 1/46 danazol courses vs 44/47 placebo courses (RR 0.023).
Tranexamic acid	Antifibrinolytic; indirect modulation of contact system	Oral	Yes	Variable (often divided dosing)	Historic controlled data: 0.34 vs 1.11 attacks/month (tranexamic acid vs placebo; RR 0.308).

Regimens reflect typical schedules from product labelling and/or pivotal trial programmes; individualization is recommended based on attack burden, patient preference, co-morbidities, and feasibility. All patients on LTP should have continued reliable access to effective on-demand therapy for breakthrough attacks.

(i) Efficacy figures are reported using the primary (or most commonly cited) endpoint from each pivotal programme and are presented for orientation only; endpoints, eligibility (baseline attack rates), run-in requirements, analysis windows, rescue rules, and statistical methods (including model-based estimates in some trials) differ between programmes, so cross-trial comparisons should be interpreted cautiously and do not constitute head-to-head evidence. (ii) Attack rates are presented as time-normalized values using the trial’s specified reporting convention (e.g. attacks per month or per 4 weeks) and, where applicable, may reflect model-based estimates rather than simple observed means. ‘Attack-free’ and ‘attacks requiring on-demand therapy’ are reported as defined in each programme and may be influenced by local treatment-seeking behaviour and rescue practices. (iii) For crossover trial designs (e.g. SC pdC1-INH), rates and attack-free outcomes relate to the trial-defined treatment periods and should be interpreted in the context of within-patient comparisons rather than parallel-group estimates. (iv) For legacy agents (attenuated androgens and antifibrinolytics), efficacy estimates (where shown) are derived from older, generally small controlled studies and long-standing real-world use with heterogeneous outcome definitions; these values are provided for historical/contextual comparison only and are not directly comparable with modern targeted prophylaxis trials.

FXIIa, activated factor XII; SC, subcutaneous, RR= relative risk.

### pdC1-INH—Cinryze and HAEGARDA

Plasma-derived C1-INH replacement reduces contact system activation upstream by inhibiting FXIIa and plasma kallikrein, thereby lowering bradykinin generation. Cinryze is administered intravenously at regular intervals (commonly every 3–4 days or twice weekly) for prophylaxis. In a randomized, double-blind, placebo-controlled crossover trial, prophylactic C1-INH significantly reduced attack frequency over 12-week periods (6.26 attacks per 12 weeks on C1-INH vs 12.73 on placebo). There were also reductions in attack severity, duration and days of swelling, and reduced need for rescue therapy (mean 4.7 vs 15.4 on-demand injections used over 12 weeks, Cinryze vs placebo). Attack-free outcomes were not reported as a defined endpoint in the pivotal publication [9].

HAEGARDA is plasma-derived C1-INH replacement therapy but designed for subcutaneous administration. This route produces different pharmacokinetics with slower absorption and more sustained plasma concentrations and avoids the need for regular IV access. It is not used for on-demand treatment. In the Phase 3 COMPACT trial, twice-weekly subcutaneous C1-INH (60 IU/kg) markedly reduced attack frequency versus placebo [32]. Extension and additional analyses support durability of benefit with generally favourable tolerability, with injection-site reactions the most common adverse event. Around 40% of patients were attack-free during the 14-week efficacy observation periods (Weeks 3–16) on the licensed 60 IU/kg twice-weekly regimen (vs 0% in the placebo period) and markedly reduced number of attacks requiring on-demand treatment (35 vs 358 total rescue-treated attacks; SC C1-INH 60 IU/kg vs placebo) [33].

### Lanadelumab (Takhzyro)

Lanadelumab is a fully human IgG1-Kappa monoclonal antibody that inhibits plasma kallikrein and is administered subcutaneously (300 mg every 2–4 weeks, depending on control and regimen). In the Phase 3 HELP trial, lanadelumab significantly reduced attack rates compared with placebo. Model-based attack rates were approximately 0.26–0.53 attacks per month on lanadelumab, versus 1.97 per month with placebo [34]. Open-label extension data support sustained reductions in attack burden with expected tolerability (injection site reactions and intercurrent viral upper respiratory tract infections commonly reported) [35]. The proportion of patients who were attack-free over 26 weeks was 44.4% with lanadelumab 300 mg Q2W, 31% with 300 mg Q4W, and 39.3% with 150 mg Q4W, compared with 2.4% with placebo. The mean monthly number of attacks requiring on-demand treatment was 0.21, 0.42, and 0.31, respectively, compared with 1.64 with placebo [34].

In HELP, low-titre anti-drug antibodies (ADAs) occurred in 11.9% of lanadelumab-treated patients (neutralizing in two patients), and in HELP OLE, ADAs were detected in 9.9% (of which 2.8% were neutralizing), with no observed impact on efficacy or safety [34, 35]. Prospective Phase 4 real-world data (EMPOWER) show sustained low attack rates with follow-up up to 36 months, arguing against clinically meaningful loss of effectiveness in routine use, and this was supported by earlier real-world data [36, 37].

### Berotralstat (Orladeyo)

Berotralstat is an oral, once-daily small-molecule inhibitor of plasma kallikrein. In the Phase 3 APeX-2 trial, berotralstat reduced investigator-confirmed attack rates at both 110 and 150 mg daily compared with placebo (approximately 1.65 and 1.31 attacks/month vs 2.35 attacks/month, respectively), with gastrointestinal adverse effects the most common driver of discontinuation in some patients [38]. Therefore, it is recommended to be taken with food to improve gastrointestinal tolerability [39]. The number of attacks requiring on-demand treatment was 1.04 versus 2.05 attacks/month (berotralstat 150 mg vs placebo). However, attack-free at 24 weeks was not meaningfully different versus placebo in the pivotal data [40, 41].

In a UK multicentre audit across 18 immunology centres (*n* = 164 HAE type 1/2), berotralstat was associated with a significant reduction in attack frequency, with median attacks falling from 4/month at baseline to 1/month at 24 months (*P* < 0.0001), alongside improved Angioedema Control Test scores. Adverse effects were reported in 49% (most commonly gastrointestinal), and 33% discontinued berotralstat during the audit observation window, most commonly due to perceived lack of efficacy (19%) or adverse effects (12%) [42].

### Garadacimab (Andembry)

Garadacimab is a fully human IgG4-Lambda monoclonal antibody that inhibits activated factor XII (FXIIa), an upstream trigger of the contact system, thereby reducing downstream plasma kallikrein and bradykinin generation. It is administered once a month as a subcutaneous injection, with an additional loading dose at initiation. Pre-licensing evidence was based on a single pivotal 6-month randomized controlled trial with a sample size of 64 entering the treatment period (39 garadacimab vs 25 placebo). In the Phase 3 VANGUARD trial, garadacimab substantially reduced the time-normalized HAE attack rate compared with placebo (0.27 vs 2.01 attacks per month, respectively), with a high proportion of participants attack-free over the treatment period (62% on garadacimab vs 0% on placebo) and an overall favourable safety profile. The number of attacks requiring on-demand treatment was 0.23 versus 1.86 attacks/month (garadacimab vs placebo) [43].

### Donidalorsen (DAWNZERA)

Donidalorsen is a GalNAc-conjugated antisense oligonucleotide designed for hepatocyte uptake that reduces prekallikrein mRNA, thereby lowering downstream plasma kallikrein activity. It is administered as a subcutaneous injection. The recommended regimen is 80 mg every 4 weeks, with 80 mg every-8-week option in selected patients [44].

In the Phase 3 OASIS-HAE randomized, double-blind, placebo-controlled trial, the primary endpoint was time-normalized investigator-confirmed HAE attack rate per 4 weeks. Donidalorsen 80 mg every 4 weeks reduced the mean 4-weekly attack rate compared with placebo (0.44 vs 2.26, respectively), and 8 weekly dosing reduced attacks to 1.02. Secondary endpoints supported clinically meaningful reductions in moderate/severe attacks (0.12 vs 1.15) and attacks requiring acute therapy (0.15 vs 1.8), and a higher attack-free proportion (53% vs 9%) on 4-weekly regimen [45].

### Attenuated androgens—danazol, stanazolol, and oxandrolone

Historic controlled trial data (small studies with heterogeneous endpoints) suggest substantial prophylactic efficacy; in a systematic review, danazol was associated with attacks in 1/46 danazol treatment courses versus 44/47 placebo courses (Relative risk [RR] 0.023) [46, 47]. These estimates come from older, small trials with non-standardized outcome definitions and should not be interpreted as directly comparable efficacy versus modern targeted prophylactic programmes. AAs increase hepatic synthesis of C1-INH and other regulatory proteins, which reduce attack frequency. They remain in use in many countries because they are inexpensive, oral and often immediately available. The main limitation is dose- and duration-dependent toxicity, including weight gain, acne, menstrual disturbance, virilization, mood changes, hypertension, dyslipidaemia, and hepatotoxicity (including rare hepatic adenoma/carcinoma risk with long-term exposure). Contemporary guidance generally recommends using the lowest effective dose and avoiding sustained high-dose regimens. Monitoring commonly includes regular blood pressure and laboratory surveillance (e.g. full blood count and liver enzymes) with periodic liver imaging. Androgens are contraindicated in pregnancy and avoided in children and in patients with significant hepatic disease or other high-risk co-morbidities [1, 2].

### Tranexamic acid

In a controlled trial summarized in the same historical systematic review, tranexamic acid reduced attack frequency to 0.34 attacks/month (32 attacks/94 patient-months) versus 1.11 attacks/month with placebo (63/57 patient-months) (RR 0.308) [46, 48]. Anti-fibrinolytics such as tranexamic acid modulate plasmin generation and downstream contact system activation; their mechanism in HAE is less direct than that of bradykinin-pathway targeted agents, and efficacy is often lower in adults than with androgens or targeted prophylaxis. However, tranexamic acid is better tolerated and has a longer history of use in children (where clinically appropriate) and remains as a bridging or fall-back LTP option globally because of low cost and wide availability. Limitations include lower efficacy and, in some patients, no efficacy, and its use in patients with thrombotic risk factors [1, 2]. There are reports of the use of tranexamic acid in pregnancy for HAE prophylaxis, but considering the low efficacy and increased risk of thromboembolism, the authors would not recommend it for this indication.

### Investigational: Deucrictibant extended release

In parallel with the immediate release version, an extended-release tablet formulation of Deucrictibant is being developed for once daily LTP, aiming to provide sustained B2-receptor antagonism. The phase 3 CHAPTER-3 trial (NCT06669754) is evaluating oral Deucrictibant extended release (XR) versus placebo for prophylaxis in adolescents and adults with HAE-C1-INH [30, 49]. Longer-term safety and durability of effect are being assessed in an ongoing open-label study of Deucrictibant XR (NCT06679881) [50].

### Investigational: Navenibart (STAR-0215)

Navenibart is an investigational long-acting monoclonal antibody inhibitor of plasma kallikrein, engineered to extend circulating half-life with the goal of substantially reduced treatment burden through infrequent subcutaneous dosing (e.g. every 3–6 months). A pivotal phase 3, randomized, placebo-controlled trial (ALPHA-ORBIT) is ongoing to evaluate efficacy and safety in adolescents and adults with HAE-C1-INH using 3–6 monthly regimes [51].

### Investigational: gene editing (Lonvo-z)

Lonvo-z (lonvoguran ziclumeran; formerly NTLA-2002) is an investigational *in vivo* CRISPR–Cas9 gene-editing therapy targeting KLKB1 in hepatocytes, intended to provide durable suppression of prekallikrein after a single infusion. This approach is categorically different from reversible pharmacological prophylaxis: rather than transiently inhibiting the kallikrein–kinin pathway, it aims to durably edit the gene encoding prekallikrein. Early clinical data have shown substantial reductions in attack frequency, but the risk–benefit assessment differs from monoclonal antibodies, small molecules, or antisense therapy because this intervention is not readily reversible once administered [52]. Long-term follow-up should therefore evaluate not only durability of attack reduction, but also off-target editing, unintended on-target editing outcomes, liver safety, immunogenicity to the editing components, and late-emerging clinical consequences of sustained prekallikrein reduction. Preclinical off-target assessment has identified intronic MAPK1 editing, with available analyses suggesting no functional consequence; nevertheless, this illustrates why continued genomic and clinical safety surveillance is central to evaluating this therapeutic class [53]. Given the availability of multiple effective, reversible prophylactic options, the role of an irreversible or near-irreversible approach should be considered carefully, including in the context of reported co-morbidities in hereditary prekallikrein (Fletcher factor) deficiency, such as infrequent reports of cardiovascular events, bleeding after surgery, and autoimmune disease [54, 55].

### Pre-procedural prophylaxis

Even in patients on LTP, additional prophylaxis is recommended before procedures that may precipitate attacks, particularly those involving airway manipulation (e.g. dental work, ENT interventions, and endoscopy with instrumentation) or major surgery. Contemporary guidance commonly favours short-term prophylaxis with C1-INH shortly before the procedure (timing and dose per local protocol and product availability), alongside a clear peri-operative plan for airway management and immediate access to effective on-demand therapy for breakthrough swelling. Where C1-INH is unavailable, some centres use AAs for several days pre-procedure and sometimes post-procedure, as a pragmatic alternative, but this approach is less preferred and may be unsuitable in pregnancy, children or where androgen toxicity is a concern [1, 2].

### Synthesis of evidence for long-term prophylaxis

The modern LTP evidence base shows substantial reductions in attack frequency with targeted prophylactic agents, but comparisons between options remain indirect. This is because most pivotal trials were placebo-controlled, used different eligibility criteria and run-in periods, and reported different outcomes and used different outcome measures. Placebo-controlled designs were important for regulatory approval but are less informative for day-to-day clinical decision-making, where the practical question is access and appropriateness of a prophylactic strategy for an individual patient. In high-resource settings, LTP selection increasingly reflects shared decision-making. Factors such as treatment burden (route, dosing frequency, adverse effect, etc.), co-morbidities, pregnancy, and continued need for on-demand therapy. In lower-resource settings, the decision is often constrained by availability and cost rather than preference, meaning that older oral agents such as AAs continue to be used because they are inexpensive, familiar, and easy to distribute. However, the lack of robust comparative evidence between AAs and modern targeted agents is a major evidence gap, particularly for cost-effectiveness discussions in health systems where targeted agents are difficult to fund. Further research is needed to compare prophylactic strategies, standardize outcome measures, evaluate long-term adherence and durability of response, expand paediatric and pregnancy safety data, and define pragmatic treatment algorithms for countries where access to therapies is limited or intermittent.

### From evidence to access: global representation in HAE treatment trials

The therapeutic evidence summarized earlier demonstrates that targeted on-demand and LTP therapies can substantially improve outcomes in HAE. However, the relevance of this evidence to global practice depends not only on efficacy, but also on where trials are conducted, which populations are represented, and whether HAE can realistically be diagnosed and effective therapies prescribed, delivered, and sustained in different health systems.

Early treatment and self-treatment models are strongly promoted in contemporary guidance, but these recommendations require reliable diagnostic infrastructure, rapid access pathways (emergency services/airway care), stable supply chains, financial accessibility particularly in resource-limited settings, aseptic technique and training for IV or injectable administration, and cold-chain storage for some agents. The same structural requirements also shape the feasibility of LTP, which depends not only on initiation but on sustained availability, repeat dispensing, and continuity of supply. In health systems where these assumptions do not hold, guideline-compliant care may be structurally unattainable despite clinician and patient intent. In this context, oral agents may reduce barriers related to administration logistics, but they will not by themselves resolve challenges of registration, procurement, affordability, and supply stability.

**Figure 2:**
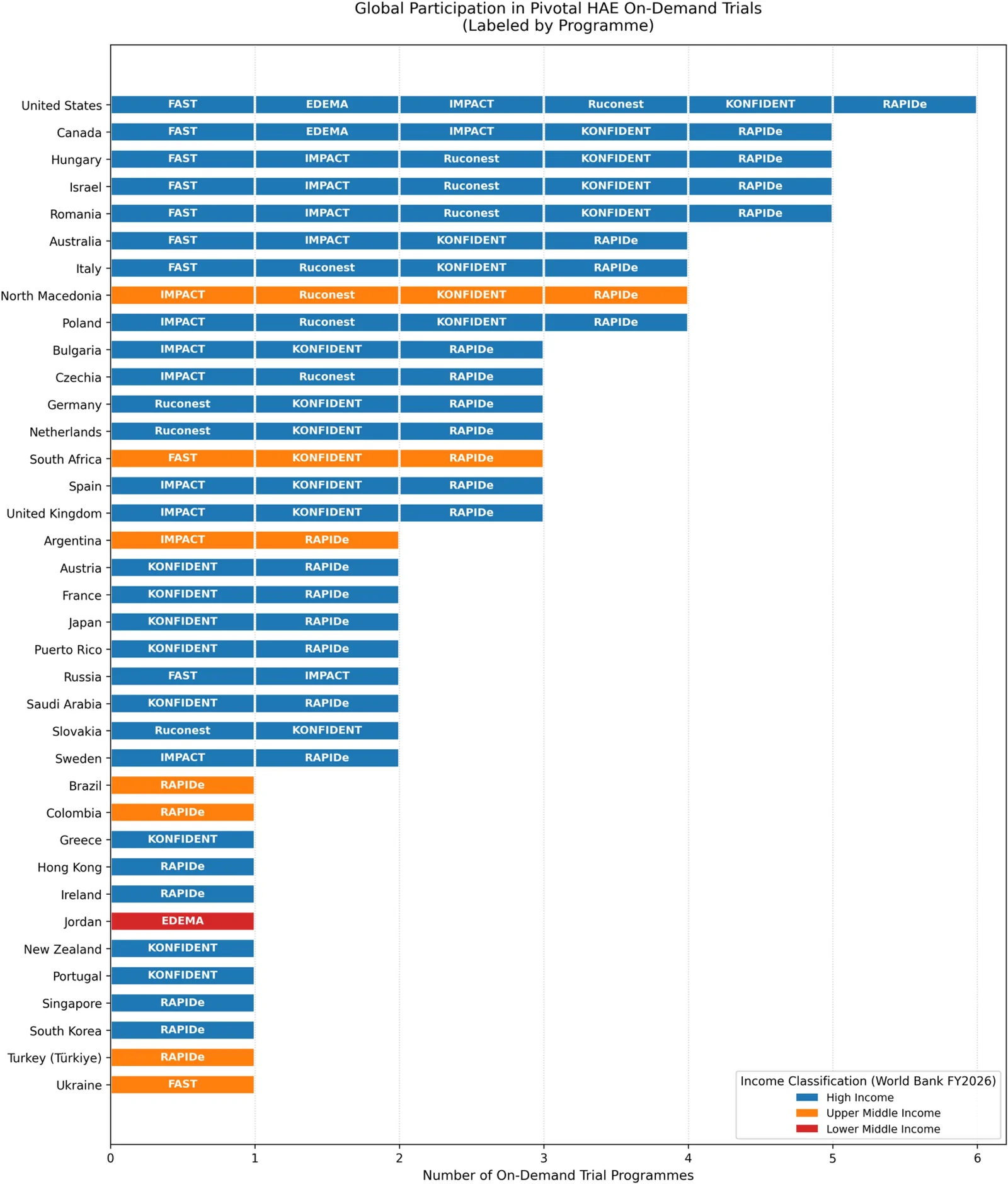
Global participation in pivotal on-demand hereditary angioedema (HAE) clinical trial programmes (*n* = 37 countries/territories). Countries are ranked in descending order by the number of distinct on-demand trial programmes in which they hosted ≥1 study site (maximum 6). Each equal-width labelled segment denotes participation in a programme: FAST (icatibant; FAST-1/2/3), EDEMA (ecallantide; EDEMA-3/4), IMPACT (pdC1-INH; Berinert; IMPACT-1/2), Ruconest (rhC1-INH; conestat alfa; C1–1102 to C1-1310), KONFIDENT (sebetralstat; KONFIDENT, KONFIDENT-S, KONFIDENT-KID), and RAPIDe (deucrictibant; RAPIDe-1/2/3). Country/territory participation was extracted from ClinicalTrials.gov study-site listings (which may be incomplete). Country-level participant numbers were not readily available. Countries/territories are colour-coded by World Bank FY2026 income classification: high-income (blue), upper-middle-income (orange), and lower-middle-income (red). Participation is concentrated in high-income settings (29/37), with one country represented across all six programmes.

Across the pivotal clinical trials for on-demand treatment, recruitment has been overwhelmingly concentrated in a small number of high-income countries (Fig. 2). Similar constraints are likely to influence participation in modern LTP programmes, although trial inclusion criteria, run-in requirements, and follow-up intensity can further limit participation in under-resourced settings. There has been repeated participation from Western/Northern European countries, and a substantial minority contribution from upper-middle-income countries such as Eastern/South-Eastern Europe and Latin America and, rarely, South Africa. In contrast, lower–middle-income and low-income countries are nearly absent from these trials. This imbalance is consistent with a broader analysis of modern HAE trial evidence base (64 trials; 4354 participants), which highlights persistent disparities within participation of clinical trials [56]. Consequently, large regions with sizeable populations are under-represented, including most of Sub-Saharan Africa (beyond South Africa), South Asia, Southeast Asia, large parts of the Middle East and North Africa (except Israel and Saudi Arabia) and much of Central America and the Caribbean (with Puerto Rico as an exception).

This skewed geography likely reflects multiple interacting structural factors. Trial participation is most feasible where specialist immunology and/or allergy centres exist with laboratories capable of providing reliable complement diagnostics (e.g. C4, C1-INH antigen and function), and, where required, genetic confirmation. Trials also require a safe infrastructure with readily available emergency airway care, and reimbursement systems that can support the high cost of investigational and commercially available targeted therapies [6]. Some of the underrepresented countries are well-resourced but remain underserved. Under-representation in resource-constrained settings could be attributed to sponsor-driven exclusion of such regions for practical reasons such as regulatory heterogeneity, infrastructure gaps, and financial risks. Addressing this imbalance requires a shift towards coordinated, sustained investment by pharmaceutical companies in these poorly represented markets.

The consequence is not only an academic lack of diversity, it can also normalize models of care that assume high-income infrastructure. Trials conducted in well-resourced settings typically rely on rapid emergency pathways, reliable cold-chain storage, and infrastructure enabling home self-administration. Clinical trials can therefore function both as a source of evidence and as a practical access mechanism, offering participating patients’ early access to novel therapies while building local clinical experience and service capability at trial sites [57]. Regions excluded from trials may miss early opportunities for capability-building as well as timely exposure to new agents. The Declaration of Helsinki (paragraph 34) also requires appropriate post-trial access arrangements for participants who may benefit [58].

Evidence from other therapeutic areas indicates that hosting trials does not reliably translate into timely post-approval access to the investigational medicinal products (IMPs) tested in those countries, raising equity concerns when interpreting global trial footprints in rare diseases. In analyses of novel drugs approved by the US Food and Drug Administration, only 14% of trial-hosting countries had physical access to all IMPs they helped test within 5 years, and only 24% of IMPs tested in countries outside the USA were accessible in all countries where they were tested, with the greatest disparities in LMICs [59, 60]. This also raises ethical concerns where LMIC populations contribute to IMP evaluation without a realistic prospect of local benefit, and where trial design choices (including comparator arms) may reflect market priorities more than local standards of care [61].

### The hidden burden of HAE globally

Diagnostic delay remains a major and preventable driver of morbidity and mortality in HAE. This is amplified where awareness is low, specialist services are scarce, and complement testing is difficult to access. Approaches that simplify sample collection, transport, and storage may help reduce some diagnostic barriers. Functional C1-INH testing using dried blood spots has been evaluated as a potential alternative to conventional functional C1-INH assays and may be particularly relevant where access to specialist complement laboratories is limited [62]. Even in Europe, analysis of data from the Icatibant Outcome Survey cohort from multiple countries showed a median diagnostic delay of 8.5 years (range 0–62 years), with longer delays in type II than type I disease (21 vs 8 years), consistent with under-use or limited availability of C1-INH assays [63]. In well-resourced settings, delays still commonly approach a decade; the UK national audit reported average diagnostic delays of 10 years for type I HAE and 18 years for type II HAE, underscoring that delayed recognition is not confined to LMIC contexts [64]. In parts of Asia-Pacific, delays may be longer. In a Chinese cohort, the mean time from first symptoms to diagnosis was 12.64 years (range 1 month to 45 years) [8]. Diagnostic delays create an artificially low apparent prevalence, as registries under-capture the true burden, and procurement and service planning may remain pessimistic (e.g. very low prevalence equates to very low priority). As an example, based on expected prevalence, India should have at least 27 000 individuals with HAE nationally, yet only around 500 cases have been diagnosed according to a recent epidemiological study from India [65], implying that the overwhelming majority remain undiagnosed and statistically invisible to care pathways and policy planning [6]. The Global Registry is an important step towards improving international visibility of HAE. Early recruitment nevertheless tended to occur where infrastructure and participating centres were already established, with 1296 patients enrolled from 29 centres, across five European countries during the initial collection period, illustrating how registry outputs can reflect underlying differences in service capacity and access [7].

Even where HAE is recognized, access to targeted on-demand therapy is inconsistent. World Allergy Organisation/European Academy of Allergy and Clinical Immunology (WAO/EAACI) guidance recommends rapid access to effective on-demand treatment, ideally enabling early self-treatment and ensuring sufficient medication to treat at least two attacks [1]. Yet, in many LMICs, first-line agents (C1-INH products, Icatibant, Ecallantide) are not registered, are unaffordable, or are unavailable through public procurement pathways. Poor access to first-line agents becomes apparent in studies where international experts are canvased for their opinion on the availability of acute therapies and compliance with medications in their countries.

For example, Bangladesh relies on FFP for acute attacks, forcing hospital-based care and transfusion dependence, whereas in Indonesia acute treatment is recorded as ‘none’, with no reliable access to targeted agents or a consistent fallback pathway, and no LTP [6]. Even in some upper-middle-income settings, partial compliance reflects restrictions that matter clinically (e.g. lack of routine home therapy, restricted access based on attack frequency, or gaps in prophylaxis). The net effect is predictable: delayed administration, reliance on emergency departments, and poorer ability to deliver best practice ‘treat early, treat at home’ care.

Where modern therapies are unavailable, FFP may function as an essential bridge, albeit with significant limitations. Real-world data from two LMIC contexts (South Africa and Iran) reported 176 acute swelling episodes in 43 patients; 98 episodes were treated with FFP (median FFP volume was 400 ml [interquartile range (IQR) 280–560]). Resolution occurred in all but two episodes (2%), with a median time to resolution of 4 hours (IQR 2–12) [27]. This demonstrates that FFP can be effective in practice. However, it also illustrates why it is an inferior substitute for targeted, rapidly deployable therapies: management requires hospital attendance and blood-product logistics, whilst adverse events are non-trivial (five transfusion reactions (5%) including one anaphylaxis). In practice, FFP often becomes a marker of system constraint rather than a chosen standard of care. It is potentially lifesaving, yet its use reflects delayed treatment and the need to use facilities that may be geographically or financially inaccessible.

The human cost of under-diagnosis and poor access to effective on-demand therapy is not theoretical. Historical mortality from laryngeal oedema was approximately 30% before targeted therapies were available, and mortality remains higher among undiagnosed patients than diagnosed patients [66]. The UK national audit (2013) likewise reinforces that HAE is potentially lethal, reporting 55 HAE-related deaths across 33 families [64].

Taken together, these data support a clear continuum of preventable harm. Delayed recognition leads to delayed access, and delayed access leads to reliance on hospital-bound or fallback therapies. These constraints translate into avoidable morbidity and, in some cases, fatal outcomes, particularly for airway attacks when timely on-demand therapy is not realistically reachable. This context highlights the potential of novel oral on-demand agents (sebetralstat, deucrictibant). Their value is not only in ‘ease of use’ in high-income settings, but also logistical feasibility, by being heat-stable, easily distributed, no sterile training required, and bypassing some infrastructure barriers that have kept modern HAE care concentrated in well-resourced systems. Closing the global gap, however, requires not just new drugs, but drugs that can survive the realities of registration, procurement, affordability, and supply continuity.

## Conclusion

The therapeutic landscape for HAE-C1-INH has changed substantially, with targeted on-demand and LTP therapies offering major improvements in attack control, treatment autonomy, and quality of life. However, the evidence base remains shaped largely by placebo-controlled trials conducted in high-resource settings, with limited direct comparative evidence, incomplete paediatric and pregnancy data, and variable representation of the populations most affected by diagnostic and treatment inequity. For clinicians and health systems, the central question is therefore not only which therapies are efficacious, but whether HAE can be diagnosed reliably and whether effective therapies can be accessed, afforded, administered, and sustained in real-world practice. Oral agents and emerging prophylactic strategies may reduce some barriers to treatment delivery, but they will not by themselves resolve gaps in awareness, diagnostics, procurement, reimbursement, and supply continuity. Improving global HAE outcomes will require timelier and more reliable diagnosis, more representative trials, post-trial access planning, comparative and cost-effectiveness evidence, and treatment models that account for the realities of both high-resource and resource-constrained health systems.

## Data Availability

No new datasets were generated or analysed for this article. Data and evidence discussed in this review are available from the published resources and public resources cited in the bibliography. Data underlying Figure 2 were extracted from ClinicalTrials.gov study-site listings and World Bank FY2026 income classifications as described in the figure caption.

## Abbreviations:

ADA: anti-drug antibody
C1-INH: C1-esterase inhibitor
DBS: dried blood spot
FFP: fresh frozen plasma
FXIIa: activated factor XII
HAE: hereditary angioedema
HAE-C1-INH: hereditary angioedema due to C1-inhibitor deficiency or dysfunction
HMWK: high-molecular-weight kininogen
IMP: investigational medicinal product
IV: intravenous
LMIC: low- and middle-income country
LTP: long-term prophylaxis
MSCS: Mean Symptom Complex Severity
pdC1-INH: plasma-derived C1-inhibitor
rhC1-INH: recombinant human C1-inhibitor
SC: subcutaneous
TOS: Treatment Outcome Score
